# The Survival Effect of Chest Wall With or Without Regional Lymphatic Radiotherapy for Breast Cancer Patients With T3~4N0M0

**DOI:** 10.3389/fonc.2021.653831

**Published:** 2021-07-12

**Authors:** Guanqiao Li, Jia Yao, Junni Chen, Baizhen Cai, Xiangying Lin, Zetan Chen, Jiawei Chen, Han Wang, Shiping Yang

**Affiliations:** ^1^ Department of Breast Surgery, Hainan General Hospital (Hainan Affiliated Hospital of Hainan Medical University), Haikou, China; ^2^ Department of Radiation Oncology, Hainan General Hospital (Hainan Affiliated Hospital of Hainan Medical University), Haikou, China; ^3^ Department of Physiology, Hainan Medical University, Haikou, China

**Keywords:** breast cancer, T3~4N0M0, regional lymph node, radiotherapy, survival

## Abstract

**Background:**

Peripheral lymphatic radiotherapy in patients with pT3N0M0 and pT4N0M0 breast cancer has been a matter of considerable debate among radiation oncologists. This is the first report in a non-Caucasian population.

**Patients and Methods:**

The study included 165 pT3N0M0 and pT4N0M0 patients. Univariate, multivariate, propensity score matching (PSM), and Kaplan-Meier analyses were conducted to evaluate the survival of patients. We also review all the literature about regional lymph nodes radiation in T3-4N0M0 patients and summarize them with tables to compare with the present study.

**Results:**

The median follow-up duration was 58.7 months. Multivariate analyses showed that advance T stage and grade were dependent poor prognostic factors for OS, DMFS, LRFS, and DFS between group A (chest wall radiation) and group B (chest wall and regional lymph nodes radiation). The overall survival (OS), disease-free survival (DFS), local relapse-free survival (LRFS), and distant metastasis-free survival (DMFS) rates were not significantly different between group A and group B. The 5-year OS rate was 92.3% *vs* 89.7% for group A and group B, respectively (P=0.819). The 5-year LRFS rate was 94.9% *vs* 94.3% for group A and group B, respectively (P=0.852). Fifty-four pairs of patients were selected after propensity score matching (PSM) analysis was conducted. There was also no significant difference between group A and group B in regard to the OS, DFS, LRFS, and DMFS rates after PSM. The patients included in previous studies were all Caucasians, and our study was focused on non-Caucasians. The cases of previous studies were 10 to 20 years ago, but our study has more recent cases. The radiotherapy techniques of previous studies were conventional, and the techniques used in our study were three-dimensional conformal radiotherapy (3DCRT) or intensity modulated radiotherapy (IMRT).

**Conclusion:**

Both our study and previous studies suggested that regional lymph nodes radiation cannot improve the survival rate for breast cancer patients with T3-4N0M0 in non-Caucasian population. Advance T stage and grade were the dependent poor prognostic factors for T3-4N0M0 patients.

## Introduction

Breast cancer is one of the most prevalent cancers worldwide. If we use the seventh edition of the AJCC cancer staging, T3N0M0 is stage IIb and T4N0M0 is stage IIIa (1). A linear relationship between the tumor diameter and incidence of positive lymph nodes was indicated when using the National Cancer Institute Surveillance, Epidemiology, and End Results (SEER) data (2). This is a rare clinical scenario and the published data on breast tumors has an incidence ranging from 0.5% to 4% (3–8).

Peripheral lymphatic radiotherapy in patients with pT3N0M0 and pT4N0M0 breast cancer has also been a matter of considerable debate among radiation oncologists owning to a lack of large-scale prospective studies and data from evidence-based medicine clinic trials. Two large studies of Alphonse G. Taghian and Jennifer Goulart assess the role of postmastectomy radiotherapy (PMRT) in node-negative breast cancer patients with tumors 5 cm or larger treated by mastectomy. However, there was no comparison of patients with or without regional lymph nodes radiation. One study in 2006 analyzed the association between locoregional recurrences and peripheral lymphatic radiotherapy. However, the 156 cases in this study were collected from 1975 to 2000. Although the study suggested that there might be an association between locoregional recurrences and peripheral lymphatic radiotherapy, the numbers of the cases are limited. We felt this was worth further investigation. Another American study that analyzed the association between locoregional recurrences and peripheral lymphatic radiotherapy focused on American patients and used NCDB analysis (9).

The National Comprehensive Cancer Network guidelines indicate that patients suffering from pT3-4N0 breast cancer should be treated with RT to the chest with or without regional lymph node irradiation (RNI) (10). There are no clear criteria on using PMRT in T3-4N0M0 patients with regional lymph node. The appropriate criteria given by the American College of Radiology for postmastectomy RT is “given the conflicting prospective and retrospective data, treatment of pT3N0 patients should continue to be highly individualized”. Currently, there are no clear criteria on using PMRT in patients with pT3N0 breast cancer (11).

Do all T3-4N0M0 patients need regional lymph node irradiation? Does irradiation of regional lymph nodes improve survival rate of T3-4N0M0 patients? These questions have always puzzled oncologists. Hence, we assessed the survival rates in T3-4N0M0 patients who underwent radiotherapy of chest wall, either with or without peripheral lymphatic radiotherapy. To our knowledge, this is the first report using non-Caucasian population.

## Patients and Methods

In this study, we included 165 pT3N0M0 and pT4N0M0 patients who were treated with radiotherapy for invasive breast cancer. These patients were admitted to Hainan General Hospital from January 2010 and December 2018. This study was approved by the institutional review board and ethics committee of Hainan General Hospital. Of the 165 patients included in this study, 78 patients (47.3%) were treated with radiation at chest wall only, and 87 patients (52.7%) were treated with radiation at chest wall and the lymph node region areas.

Postmastectomy radiotherapy was used five times a week to the chest wall, supra/infraclavicular, and levels II, III axillary nodal region. There was no irradiation to the level I axilla or internal mammary chain. Patients had conventional fractionated radiotherapy, which consisted of postmastectomy radiotherapy at 50 Gy in 25 fractions over 5 weeks. The CW ± RNI was delivered using 3DCRT or IMRT.

Only one patient (0.6%) whose histopathology was pathologically mucinous adenocarcinoma did not receive chemotherapy, whereas all the other patients (99.4%) received chemotherapy. Because all the patients were lymph node-negative and had similar stages, the chemotherapy regimen was nearly the same [2 patients (1.2%) received chemotherapy regime of four-cycle TC, 1 patient (0.6%) received chemotherapy regime of four-cycle AC, and the others (98.2%) received chemotherapy regime of eight-cycle AC-T].

We analyzed the baseline characteristics and treatment patterns. The seventh American Joint Committee on Cancer (AJCC) staging system was used to assess the pathological tumor stage (1). The patients had a check in with the hospital every 3 months in the first two years, this changed to every six months for 3-5 years and once a year after that. The date of commencement of operation was set as the start date to measure events. We defined the end points (time to the first defining event) as follows: overall survival (OS), disease-free survival (DFS), local relapse-free survival (LRFS), and distant metastasis-free survival (DMFS).

### Statistical Analysis

Pearson’s chi-square test was used to compare the clinic-pathological parameters of the different groups; univariate Cox regression analysis was used to explore the risk factors of survival outcome. Using the Cox proportional hazard model, multivariate analysis of each prognostic variable was analyzed by the backward stepwise (likelihood ratio) procedure. In addition to balance of baseline characteristics between each group, propensity score matching (PSM) analysis was conducted with a ratio of 1.0. The Kaplan-Meier method was used to evaluate the survival of patients before and after matching. The difference between the two groups in survival time was investigated using log-rank test. P values less than 0.05 was considered statistically significant. Hazard ratios (HR) were presented with 95% confidence intervals. SPSS for Windows (version 22.0, SPSS Inc., Chicago, IL, USA) was used for all statistical analyses.

## Results

### Patient Baseline Characteristics According to Radiation Area

We identified 78 patients with chest wall radiation and 87 patients with chest wall + regional lymph nodes area radiation that were treated in our department. The clinico-pathologic characteristics of these patients are summarized in **Table 1**. Their average age was 48.0 years, and the median age was 47.2 years (range, 20.8–71.4 years). All the patients are female. All of them underwent surgery and chemotherapy. 87.5% of the HER2 positive patients received targeted therapy. We divided the patients into group A (chest wall radiation) and group B (chest wall + regional lymph nodes radiation) according to the RT fields. Group B had a significantly higher proportion with pre-menopausal state than group A (P=0.003). The axillary management was significantly different between the two groups. The sentinel lymph node biopsy (SLNB) was higher in group B than group A (P=0.006). There were no significant differences in T stage and histological grade between two groups. In addition, the two treatment groups were well balanced in terms of age, ER status, PR status, Her2 status, pathology, histological grade, trastuzumab, lymphovascular invasion (LVI), RT technique, and the number of negative lymph nodes.

**Table 1:** Baseline characteristics and treatment patterns for chest wall ± regional LN RT.

	Chest wall (n = 78)	chest wall+regional LN (n = 87)	P
**Age (year)**			0.271
≤40	15 (19.2%)	24 (27.6%)	
>40	63 (80.8%)	63 (72.4%)	
**Menopausal status**			0.003
Pre-menopausal	42 (53.8%)	66 (75.9%)	
Post-menopausal	36 (46.2%)	21 (24.1%)	
**ER status**			0.531
Negative	45 (57.7%)	45 (51.7%)	
Positive	33 (42.3%)	42 (51.7%)	
**PR status**			1.000
Negative	51 (65.4%)	57 (62.6%)	
Positive	27 (34.6%)	30 (37.4%)	
**Her-2 status**			0.213
Negative	48 (61.5%)	45 (65.5%)	
Positive	30 (38.5%)	42 (48.3%)	
**T Stage**			0.125
T3	63 (80.8%)	78 (89.7%)	
T4	15 (19.2%)	9 (10.3%)	
**Pathology**			0.160
Ductal	69 (88.5%)	78 (89.7%)	
Mucinous	6 (7.7%)	2 (2.3%)	
Others	3 (3.8%)	7 (8.0%)	
**Axillary management**			0.006
SLNB	3 (3.8%)	15 (17.2%)	
ALND	75 (96.2%)	72 (82.8%)	
**Negative lymph nodes (n)**			0.075
**≤5**	4 (5.1%)	7 (8.0%)	
**6**–**10**	4 (5.1%)	13 (14.9%)	
**≥11**	70 (89.7%)	67 (77.0%)	
**Histological grade**			0.909
I	9 (11.5%)	9 (10.3%)	
II	59 (75.6%)	65 (74.7%)	
III	10 (12.8%)	13 (14.9%)	
**Trastuzumab**			0.334
No	51 (65.4%)	51 (58.6%)	
Yes	27 (34.6%)	36 (41.4%)	
**Chemotherapy**			0.473
No	1 (1.3%)	0 (0%)	
Yes	77 (98.7%)	88 (100%)	
**RT technique**			0.685
3DCRT	2 (2.6%)	4 (4.6%)	
IMRT	76 (97.4%)	83 (95.4%)	
**LVI**			0.750
No	74 (94.9%)	81 (93.1%)	
Yes	4 (5.1%)	6 (6.9%)	

LN, lymph node; RT, radiotherapy; ER, estrogen receptor; PR, progesterone receptor; SLNB, sentinel lymph node biopsy; ALND, axillary lymph node dissection; LVI, lymphovascular invasion.

### Analysis of Prognostic Factors for Breast Cancer Patients

To identify which factors affect the prognosis, univariate and multivariate Cox regression analyses were performed. Univariate Cox regression analysis in **Table 2** revealed that T stage was a significant predictive factor for OS, LRFS, DMFS, and DFS. Tumor grade was a significant predictive factor for OS, DMFS, and DFS. ER was a significant predictive factor for DMFS and DFS. The OS, LRFS, DMFS, or DFS of patients with or without regional lymph nodes radiation had no significant difference.

**Table 2:** Univariate Cox regression analysis of prognostic factors.

Variables	OS		LRFS		DMFS		DFS		
	HR (95% CI)	P	HR (95% CI)	P	HR (95% CI)	P	HR (95% CI)	P
**Regional LN RT**								
No *vs* Yes	1.128 (0.401–3.781)	0.819	0.882 (0.236–3.292)	0.852	1.087 (0.386–3.064)	0.874	1.133 (0.438–2.930)	0.797
**Years**								
≤40 *vs* >40	0.418 (0.148–1.183)	0.100	0.576 (0.143–2.323)	0.438	0.444 (0.157–1.255)	0.126	0.600 (0.225–1.605)	0.309
**Menopausal status**								
Pre *vs* Post	0.549 (0.155–1.949)	0.354	2.835 (0.760–10.577)	0.121	0.545 (0.153–1.932)	0.347	0.822 (0.293–2.308)	0.710
**ER status**								
Negative *vs* Positive	0.292 (0.082–1.036)	0.057	0.914 (0.244–3.425)	0.894	0.282 (0.080–1.003)	0.005	0.314 (0.103–0.954)	0.041
**PR status**								
Negative *vs* Positive	0.489 (0.138–1.738)	0.269	1.581 (0.422–5.917)	0.497	0.486 (0.137–1.726)	0.265	0.540 (0.178–1.164)	0.278
**Her-2 status**								
Negative *vs* Positive	0.394 (0.110–1.409)	0.152	1.258 (0.333–4.744)	0.735	0.358 (0.101–1.277)	0.113	0.400 (0.131–1.222)	0.108
**T stage**								
T3 *vs* T4	4.168 (1.480–11.732)	0.007	13.003 (3.246–52.093)	0.000	4.371(1.553–12.302)	0.005	3.258 (1.221–8.694)	0.018
**Negative lymph nodes (n)**								
≤5 vs 6~10 *vs* ≥11	1.017 (0.383 - 2.703)	0.972	10.751 (0.032–66.813)	0.425	1.039 (0.391–2.760)	0.939	1.215 (0.457–3.231)	0.696
**Histopathology**								
IDC *vs* MC *vs* others	1.923 (0.977–3.787)	0.059	0.129 (0.000–71.848)	0.526	1.906 (0.971–3.740)	0.061	1.680 (0.867–3.257)	0.125
**Grade**								
I *vs* II *vs* III	2.991 (1.153–7.755)	0.024	2.604 (0.757–8.954)	0.129	2.937 (1.136–7.593)	0.026	3.623 (1.532–8.567)	0.003
**Chemotherapy**								
No *vs* Yes	20.283 (0.00–121.180)	0.914	20.301 (0.00–290.833)	0.936	20.293 (0.00–542.617)	0.876	20.296 (0.00–741.215)	0.860
**LVI status**								
No *vs* Yes	0.755 (0.099–5.770)	0.786	0.043 (0.000–73.432)	0.542	0.782 (0.102–5.979)	0.813	0.564 (0.088–5.009)	0.591
**RT technique**								
3DRT *vs* IMRT	22.408 (0.001–414.91)	0.538	21.93 (0.002–284.96)	0.523	22.036 (0.000–669.39)	0.631	21.887 (0.003–144.81)	0.492

OS, overall survival; LRFS, local relapse-free survival; DMFS, distant metastasis-free survival; FFS, failure-free survival; LVI, lymphovascular invasion; LN, lymph node; RT, radiotherapy; ER, estrogen receptor; PR, progesterone receptor.

Next, we conducted multivariate analyses to evaluate the prognostic value of T stage, histological grade, ER, and regional lymph nodes radiation (**Table 3**). T4 stage was an independent poor prognostic factor for OS (P=0.002), DMFS (P=0.006), LRFS (P=0.002), and DFS (P=0.013). Furthermore, advance grade were an independent poor prognostic factor for OS(P=0.021), DMFS (P=0.030), LRFS (P=0.045), and DFS (P=0.004).

**Table 3:** Multivariate cox regression analysis of prognostic factors.

Variables	OS		DMFS		LRFS		DFS	
	HR (95% CI)	P	HR (95% CI)	P	HR (95% CI)	P	HR (95% CI)	P
**Regional LN RT**								
No *vs* Yes	1.380 (0.453–4.203)	0.571	1.177 (0.425–3.257)	0.754	1.286 (0.318–5.199)	0.724	1.123 (0.437–2.890)	0.809
**ER status**								
Negative *vs* Positive	0.291 (0.081–1.046)	0.059	0.375 (0.122–1.154)	0.087	0.885 (0.229–3.422)	0.859	0.405 (0.1471–1.118)	0.081
**T stage**								
T3 *vs* T4	6.165 (1.920–19.798)	0.002	4.455 (1.526–13.002)	0.006	28.007 (3.505–224.771)	0.002	3.752 (1.3222–10.647)	0.013
**Grade**								
I *vs* II *vs* III	4.360 (1.315–14.454)	0.021	3.288 (1.124–9.613)	0.030	10.839 (1.058–111.037)	0.045	4.038 (1.548–10.531)	0.004

OS, overall survival; LRFS, local relapse-free survival; DMFS, distant metastasis-free survival; FFS, failure-free survival; LN, lymph node; RT, radiotherapy; ER, estrogen receptor.

### Survival Analysis

**Figure 1:**
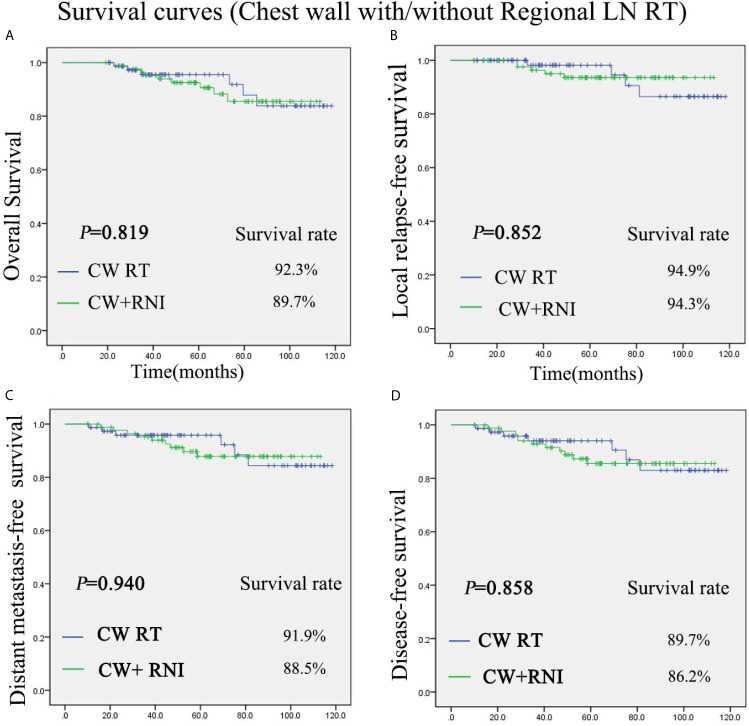
Kaplan-Meier survival curves for chest wall radiation group and chest wall + regional lymph nodes radiation group. Overall survival **(A)**, disease-free survival **(B)**, local relapse-free survival **(C)**, and distant metastasis-free survival **(D)**. P values were calculated with the unadjusted log-rank test. CW, chest wall; RNI, regional lymph node irradiation.

The median follow-up duration for the patients (range, 19.2–118.3 months) was 58.7 months. There was no significant difference between group A (chest wall radiation) and group B (chest wall and regional lymph nodes radiation) in regard to the OS, DFS, LRFS, and DMFS rates. The 5-year OS rate was 92.3% *vs* 89.7% for the group A and group B, respectively (P = 0.819) (**Figure 1A**). The 5-year LRFS rate was 94.9% *vs* 94.3% for the group A and group B, respectively (P = 0.852) (**Figure 1B**). For group A, the 5-year DMFS rate was 91.9%. For group B, it was 88.5% (P = 0.940) (**Figure 1C**). The 5-year DFS rates of group A was 89.7%, and group B was 86.2% (P = 0.858) (**Figure 1D**).

In addition, some baseline characteristics were significant difference between group A (chest wall radiation) and group B (chest wall and regional lymph nodes radiation). To balance of baseline characteristics between each group, propensity score matching (PSM) analysis was conducted. Fifty-four pairs of patients were selected for analysis. The clinico-pathologic characteristics after matching are summarized in **Table 4**. The baseline characteristics of two treatment groups were well balanced after matching.

**Table 4:** Baseline characteristics and treatment patterns for chest wall ± regional LN RT after propensity score matching.

	chest wall (n = 54)	chest wall+regional LN (n = 54)	P
**Age (year)**			0.628
≤40	12 (22.2%)	9 (16.7%)	
>40	42 (77.8%)	45 (83.3%)	
**Menopausal status**			0.418
Pre-menopausal	33 (61.1%)	38 (70.4%)	
Post-menopausal	21 (38.9%)	16 (29.6%)	
**ER status**			0.694
Negative	34 (63.0%)	31 (57.4%)	
Positive	20 (37.0%)	23 (42.6%)	
**PR status**			1.000
Negative	40 (74.1%)	39 (72.2%)	
Positive	14 (25.9%)	15 (27.8%)	
**Her-2 status**			0.847
Negative	30 (55.6%)	28 (51.9%)	
Positive	24 (44.4%)	26 (48.1%)	
**T Stage**			1.000
T3	45 (83.3%)	45 (83.3%)	
T4	9 (16.7%)	9 (16.7%)	
**Pathology**			1.000
Ductal	54 (100%)	54 (100%)	
Others	0 (0%)	0 (0%)	
**Axillary management**			1.000
SLNB	3 (5.6%)	3 (5.6%)	
ALND	51 (94.4%)	51 (94.4%)	
**Negative lymph nodes (n)**			0.761
0–10	7 (13.0%)	5 (9.3%)	
≥11	47 (87.0%)	49 (90.7%)	
**Histological grade**			1.000
I∼II	49 (90.7%)	49 (90.7%)	
III	5 (9.3%)	5 (9.3%)	
**Chemotherapy**			1.000
No	0 (0%)	0 (0%)	
Yes	54 (100%)	54 (100%)	
**Trastuzumab**			0.845
No	33 (61.1%)	31 (56.6%)	
Yes	21 (38.9%)	23 (41.4%)	
**LVI**			0.118
No	50 (92.6%)	54 (100%)	
Yes	4 (7.4%)	0 (0%)	

LN, lymph node; RT, radiotherapy; ER, estrogen receptor; PR, progesterone receptor; SLNB, sentinel lymph node biopsy; ALND, axillary lymph node dissection; LVI, lymphovascular invasion.

**Figure 2:**
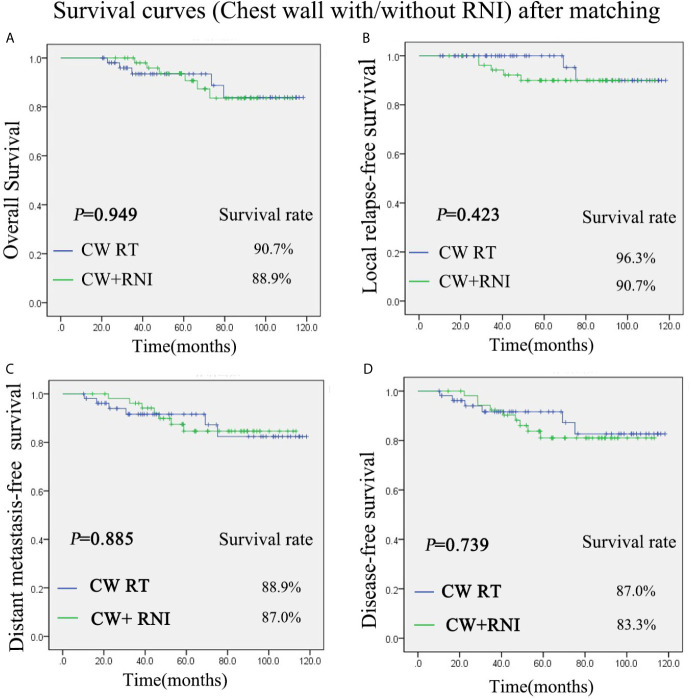
Kaplan-Meier survival curves after propensity score matching (PMS) for group of chest wall radiation and group of chest wall + regional lymph nodes radiation. Overall survival **(A)**, disease-free survival **(B)**, local relapse-free survival **(C)**, and distant metastasis-free survival **(D)**. P values were calculated with the unadjusted log-rank test. CW, chest wall; RNI, regional lymph node irradiation.

There was also no significant difference between group A (chest wall radiation) and group B (chest wall and regional lymph nodes radiation) in regard to the OS, DFS, LRFS, and DMFS rates after PSM. The 5-year OS rate was 90.7% *vs* 88.9% for the group A and group B, respectively (P = 0.949) (**Figure 2A**). The 5-year LRFS rate was 96.3% *vs* 90.7% for the group A and group B, respectively (P = 0.423) (**Figure 2B**). For group A, the 5-year DMFS rate was 88.9%. For group B, it was 87.0% (P = 0.885) (**Figure 2C**). The 5-year DFS rates of group A was 87.0%, and group B was 83.3% (P = 0.739) (**Figure 2D**).

## Discussion

The T3-4N0M0 breast cancer is relatively rare, so it is difficult to carry out a phase III clinical trial study. We repeatedly reviewed the extensive literature on this topic by PubMed. We searched PubMed with the Title/Abstract of “Breast cancer, T3N0M0 (or T4N0M0), radiotherapy” published from 1990/01/01 to 2020/10/01. There were only four relevant publications. We summarized and compared the related literature of the previous four studies and our current research, and the results are shown in **Tables 5**, **6**. As shown in **Table 5**, the collection time of the four previous studies (5, 9, 12, 13) is relatively long (10 to 20 years ago), and the cases in our study all within the last 10 years. The patients of previous studies were all Caucasians, and we conducted this study using non-Caucasian patients. Besides, the number of cases in the other three retrospective studies is relatively small and most are postoperative radiotherapy cases, except the Cassidy RJ’s study (9), which was an NCDB analysis from the United States. Two of them (5, 12) only listed the number of regional lymph nodes radiation cases but did not carry out survival analysis of patients, whereas the other carried out OS comparative analysis in the subgroup analysis of NCDB study in the United States. The advantage of NCDB study is the large numbers of patients. However, data information of NCDB is often incomplete. 16% of the cases in Cassidy RJ’s research (9) lack ER and PR status information, and 75% of the cases lack HER2 status. Moreover, detailed information on chemotherapy and hormone therapy were unavailable. The retrospective study of G.Aksu (13) collected the information of patients from 1975 to 2000 and lacked the status information of ER/PR or HER2. The radiation technology used in the study could now be considered outdated. The chest wall and/or peripheral lymphatics were administered with two daily fractions by Co60-6MV photon beam and electron beams after mastectomy. In our study, the information of each patient is relatively complete, and the radiation technology is based on modern radiotherapy techniques (3DCRT or IMRT). IMRT technology reduced the contralateral breast, ipsilateral lung, skin toxicity, and maintained reasonable target homogeneity when compared with conventional tangential techniques (14–16). The radiotherapy techniques of the previous study were conventional, and the techniques used in our study were 3DCRT or IMRT. Our results suggested that increasing regional lymph node irradiation did not improve the overall survival rate, which is consistent with the results of Cassidy RJ (9) and G.Aksu (13), whereas the Cassidy RJ study did not analyze the regional lymph node irradiation on recurrence rate (**Table 6**). Our study also analyzed the local recurrence rate. The results suggested that increasing regional lymph node radiation did not reduce the local recurrence rate.

**Table 5:** Characteristics on radiotherapy to chest wall with or without regional lymph node in previous three reports and the present study.

Author	Country	Published time	NO. of Cases		Date of cases	PMRT	CW	CW+RNI	Radiotherapy techniques
						(NO.)	(NO.)	(NO.)	
			T3	T4					
**Cassidy RJ** (9)	America	2017	3437 NCDB	0	2003–2011	1644	1094	550	No documented
**Goulart J** (12)	Canada	2011	100	0	1989–2000	44	25	19	No documented
**G. Aksu** (13)	Turkey	2007	133	23	1975–2000	147	50	66	Conventional techniques
**Helinto M** (5)	Finland	1999	38	0	1987–1994	33	29	4	Conventional techniques
**Present study**	China	/	141	24	2010–2018	165	78	87	3DCRT or IMRT

NO., number; PMRT, postmastectomy radiotherapy; CW, chest wall; RNI, regional lymph node irradiation; 3DCRT, three-dimensional conformal radiotherapy; IMRT, intensity modulated radiotherapy.

**Table 6:** Survival radiotherapy to chest wall with or without regional lymph node in previous three reports and the present study.

Author	Median follow-up	OS	DFS	LRFS	DMFS	Suggestion
	(month)	(CW *vs.* CW+RNI)	(CW *vs.* CW+RNI)	(CW *vs.* CW+RNI)	(CW *vs.* CW+RNI)	
**Cassidy RJ** (9)	55.7	87.9% *vs.* 85.6%	/	/	/	RNI may not be needed
		P=0.06				
**Goulart J** (12)	120.0	/	/	/	/	/
**G. Aksu** (13)	90.0	85.0% *vs.* 65.0%	/	93.0% *vs.* 87.0%	/	RNI should be neglected
		P=0.26		NS		
**Helinto M (** 5)	58.0	/	/	/	/	/
**Present study**	58.7	92.3% *vs.* 89.7% P=0.819	89.7% *vs* 86.2% P=0.858	94.9% *vs* 94.3% P=0.852	91.9% *vs* 88.5% P=0.940	RNI may not be needed

CW, chest wall; RNI, regional lymph node irradiation; OS, overall survival; LRFS, local relapse-free survival; DMFS, distant metastasis-free survival; FFS, failure-free survival.

Taken together, our results are consistent with previous results. We also analyzed the distant metastasis and disease-free survival rate. The results showed that there was no significant difference in distant metastasis and disease-free survival rate between chest wall + regional lymph node irradiation and chest wall radiation only. Therefore, for T3-4N0M0 patients, we do not recommend radiation of regional lymph nodes. Our research also indicates that advance T stage and grade were poor prognostic factors for OS, DMFS, LRFS, and DFS. Will advance T stage and grade profit from radiation of regional lymph nodes? Because of the small sample size in this study, we did not carry out further subgroup analysis.

The limitations of the study are that it is a retrospective study and the follow-up time was not long enough. In addition, the scale of cases is relatively small because T3-4N0M0 is a rare case, making large-scale case research is difficult to achieve.

## Conclusion

Both our study and previous studies suggested that regional lymph nodes radiation cannot improve the survival rate for breast cancer patients with T3-4N0M0 in a non-Caucasian population. We suggest that regional lymph nodes radiation might be neglected. Advance T stage and grade were the dependent poor prognostic factors for T3-4N0M0 patients.

## Data Availability Statement

The original contributions presented in the study are included in the article/supplementary material. Further inquiries can be directed to the corresponding authors.

## Ethics Statement

The studies involving human participants were reviewed and approved by Hainan General Hospital. Written informed consent for participation was not required for this study in accordance with the national legislation and the institutional requirements.

## Author Contributions

SY designed and edited the manuscript of the study. LG and SY collected and interpreted the data, and drafted the manuscript. JY and HW carried out statistical analysis and critically revised the manuscript. JNC, BC, JWC, and XL were involved in study design, statistical analysis, data interpretation. All authors contributed to the article and approved the submitted version.

## Funding

This work was supported by Natural Science Foundation of Hainan Province, China (819QN345) and (818QN314).

## Conflict of Interest

The authors declare that the research was conducted in the absence of any commercial or financial relationships that could be construed as a potential conflict of interest.

## Abbreviations

LN, lymph node; RT, radiotherapy; ER, estrogen receptor; PR, progesterone receptor; SLNB, sentinel lymph node biopsy; ALND, axillary lymph node dissection; LVI, lymphovascular invasion; OS, overall survival; LRFS, local relapse-free survival; DMFS, distant metastasis-free survival; DFS, disease-free survival; PSM, propensity score matching.
